# Frozen versus fresh embryo transfer on perinatal outcomes—do endometrial preparation methods matter?

**DOI:** 10.1093/hropen/hoag002

**Published:** 2026-01-12

**Authors:** Haowen Zou, Deirdre Zander-Fox, Nicole Au, Yanhe Liu, Beverley Vollenhoven, Mark P Green, Rui Wang

**Affiliations:** Department of Obstetrics and Gynaecology, Monash University, Melbourne, Victoria, Australia; Monash IVF Group, Cremorne, Victoria, Australia; Biomedical Discovery Institute, Monash University, Melbourne, Victoria, Australia; Department of Biomedicine, University of Adelaide, Adelaide, Australia; Department of Obstetrics and Gynaecology, Monash University, Melbourne, Victoria, Australia; Fertility North, Perth, Western Australia, Australia; School of Human Sciences, The University of Western Australia, Perth, Western Australia, Australia; Department of Obstetrics and Gynaecology, Monash University, Melbourne, Victoria, Australia; Monash IVF Group, Cremorne, Victoria, Australia; Women’s and Newborn Program, Monash Health, Melbourne, Victoria, Australia; Monash IVF Group, Cremorne, Victoria, Australia; School of BioSciences, University of Melbourne, Melbourne, Victoria, Australia; Department of Obstetrics and Gynaecology, Monash University, Melbourne, Victoria, Australia; NHMRC Clinical Trials Centre, Faculty of Medicine and Health, University of Sydney, Sydney, New South Wales, Australia

**Keywords:** frozen embryo transfer, fresh embryo transfer, endometrium preparation, hormone replacement treatment, natural cycle, vitrification, birthweight, gestational age, preterm birth, caesarean section

## Abstract

**STUDY QUESTION:**

Are there differences between perinatal outcomes following frozen versus fresh embryo transfer in IVF, and do endometrium preparation methods contribute to the differences?

**SUMMARY ANSWER:**

Compared with fresh embryo transfers, frozen transfers, regardless of hormone replacement treatment or natural treatment cycles, were associated with lower chances of preterm birth, low birth weight, and small for gestational age, but higher chances of caesarean section, high birth weight, and large for gestational age.

**WHAT IS KNOWN ALREADY:**

Frozen embryo transfer has been increasing over the past two decades, but its associated perinatal risks and underlying reasons remain controversial. Most existing observational studies have not accounted for multiple cycles from the same couple or known patients’ characteristics or treatment protocols, such as endometrial preparation methods, in the analysis. Existing birthweight centile charts are likely to underestimate intra-uterine growth restriction due to the inclusion of deliveries following obstetric interventions.

**STUDY DESIGN, SIZE, DURATION:**

This multicentre retrospective cohort study used routinely collected clinical data of 8081 women undergoing IVF who gave birth to 9243 babies (6125 from the frozen and 3118 from fresh transfer cycles) in 12 IVF clinics across two states in Australia between 2015 and 2021, with follow-up data up to 2023.

**PARTICIPANTS/MATERIALS, SETTING, METHODS:**

Individuals undergoing autologous-oocyte IVF cycles who had singleton live births were included. An individual could have multiple treatment cycles included. Perinatal outcomes included preterm birth, low/high birth weight, small-/large-for-gestational-age, and caesarean section. The birthweight percentiles were calculated based on the New Australian birthweight centiles, where interventions-initiated births were excluded. Multivariable Poisson regression with robust variance was used to analyse all outcomes. Generalized estimating equations (GEEs) were used to account for the cluster effects of multiple embryo transfer cycles of the same individual. Adjusted risk ratios with 95% confidence intervals (CIs) were reported for each outcome. The adjusted model accounted for potential confounding factors, including female age, parity, semen source, ovulatory disorders, preimplantation genetic testing for aneuploidy, blastocyst transfer, number of embryos transferred, and site. In subgroup analysis, frozen transfers with different endometrial preparation methods (hormone replacement and natural cycles) were compared to the fresh transfers.

**MAIN RESULTS AND THE ROLE OF CHANCE:**

Compared with births in the fresh group, births in the frozen transfer group were less likely to be preterm (8.9% vs 13.7%, adjusted risk ratio (aRR) 0.66 0.58–0.76), low birth weight (5.3% vs 8.5%, aRR 0.66, 0.55–0.78), and small for gestational age (4.2% vs 7.9%, aRR 0.62, 0.51–0.75), but more likely to be caesarean section (57.5% vs 50.4%, aRR 1.14, 95% CI 1.09–1.19), high birth weight (10.1% vs 7.0%, aRR 1.43, 1.21–1.68), and large-for-gestational-age (20.0% vs 13.6%, aRR 1.37, 1.23–1.53). The differences remained consistent when comparing hormone replacement or natural cycle frozen transfers to fresh transfers.

**LIMITATIONS, REASONS FOR CAUTION:**

The retrospective nature introduces inherent challenges of residual confounding.

**WIDER IMPLICATIONS OF THE FINDINGS:**

The results indicate that the differences in birth weight related to perinatal outcomes between frozen versus fresh embryo transfer cycles are unlikely due to the differences in the use of endometrial preparation protocols in frozen cycles, but may be attributed to other factors including impaired endometrial characteristics in fresh cycles or the process of vitrification and warming embryos in frozen cycles.

**STUDY FUNDING/COMPETING INTEREST(S):**

H.Z. was supported by a Monash Research Scholarship. N.A. was supported by an Australian Government Research Training Program Scholarship. R.W. was supported by an NHMRC Emerging Leadership Investigator grant (2009767). D.Z. and M.G. reported that they are employees of the company from which the data were analysed for this study. No conflicts of interest from other authors were reported.

**TRIAL REGISTRATION NUMBER:**

N/A.

WHAT DOES THIS MEAN FOR PATIENTS? Frozen embryo transfers have become more common in IVF than fresh embryo transfers, but there is still debate about how they affect birth outcomes. It is also unclear whether the way the uterus is prepared for the frozen cycle would make a difference.

Researchers in this study looked at over 9000 babies born to more than 8000 people who had IVF in Australia. They found that using frozen embryos reduces the risk of premature birth and having a smaller baby, but could increase the chances of delivering a larger baby and needing a caesarean section. These differences do not seem to depend on how the uterus is prepared for frozen cycles. This means other factors, such as how embryos are handled during freezing, might be more important. This information can help patients and clinicians weigh the pros and cons when planning their IVF treatment.

## Introduction

Frozen embryo transfer (FET) is increasingly used in IVF treatment to avoid compromised endometrial receptivity in the fresh cycles, to prevent the risk of late-onset ovarian hyperstimulation syndrome (OHSS), and to reduce the risk of early-onset OHSS when a gonadotropin-releasing hormone (GnRH) agonist trigger is used (Evans *et al.*, 2014; Blockeel *et al.*, 2019). Also, the increasing use of preimplantation genetic testing prompts the frozen cycles. The advanced freezing techniques of vitrification and the wide use of the elective ‘freeze-all’ strategy further boost the uptake of FET globally (Roque *et al.*, 2015; Rienzi *et al.*, 2017). Previous research has indicated that in a fresh transfer cycle, asynchronous development between the endometrium and embryo due to ovarian stimulation may undermine the potential of implantation (Bergh and Navot, 1992). To support endometrial development and to optimize endometrial receptivity, multiple endometrium preparation methods are currently employed for individuals undergoing frozen embryo transfer, but the optimal method remains unclear (Zhang *et al.*, 2023).

Evidence of differences in perinatal outcomes following frozen versus fresh embryo transfers remains controversial. Such a research question for perinatal outcomes cannot be adequately addressed in randomized controlled trials, as large trials are generally underpowered for these outcomes, due to the limited number of adverse events (Chen *et al.*, 2016; Zaat *et al.*, 2021). A meta-analysis based on observational studies suggested that frozen embryo transfer was associated with a lower risk of small for gestational age (SGA) infants and preterm delivery, but a higher risk of large for gestational age infants compared with fresh embryo transfers (Maheshwari *et al.*, 2018). However, these findings were not always consistent with those in subsequent large population-based cohort studies (Vidal *et al.*, 2017; Smith *et al.*, 2019; Westvik-Johari *et al.*, 2021; Raja *et al.*, 2022).

Notably, most of these cohort studies were conducted on cycle-based data and did not account for multiple cycles from the same couple when investigating the research question. While some national registry-based studies were able to link multiple cycles of the same couple, they were not able to account for known patients’ characteristics or clinical protocols (e.g. endometrial preparation methods), due to the absence of these clinical data in the registries (Vidal *et al.*, 2017; Smith *et al.*, 2019; Westvik-Johari *et al.*, 2021; Raja *et al.*, 2022). Thus, there is a need for large observational studies based on the routinely collected clinical data with rich information on patients’ characteristics and details on treatments to address this knowledge gap.

In addition, most existing birthweight centile charts in perinatal research are likely to underestimate intra-uterine growth restriction, due to the inclusion of deliveries following obstetric interventions, especially preterm deliveries (Joseph *et al.*, 2020). As babies following iatrogenic preterm births tended to have lower birthweights for their gestational age, including them in the reference population has lowered the corresponding birthweight centiles, resulting in a missed diagnosis of SGA for some babies at risk of growth restriction (Dobbins *et al.*, 2012). It is therefore crucial to use a birthweight centile chart that excludes intervention-initiated births to provide more accurate evidence of growth assessment.

Therefore, we performed a large multicentre observational study to investigate the association between transfer methods and perinatal outcomes, as well as the impact of different endometrial preparation methods on the differences in perinatal outcomes between frozen and fresh embryo transfer strategies. In addition, we also evaluated the impact of using the new Australian birthweight centiles where interventions-initiated births were excluded (Joseph *et al.*, 2020), as compared to the old birthweight centiles (Dobbins *et al.*, 2012).

## Materials and methods

### Study design and setting

This study was a multicentre retrospective cohort study, including all 12 IVF clinics of Monash IVF across two states (Victoria and Queensland) in Australia. The project proposal was approved by the Monash IVF research committee (2023-P12) and ethics approval was obtained from the Monash University Human Research Ethics Committee (2024-38798-104069). The Strengthening the Reporting of Observational Studies in Epidemiology (STROBE) Statement was followed when reporting this observational study (von Elm *et al.*, 2014).

### Participants

Data of individuals starting their autologous-oocyte IVF cycles between April 2015 and September 2021 was collected and cycles resulting in singleton live births were included. The exclusion criteria included: (i) non-IVF cycles (artificial insemination or timed intercourse cycles); (ii) vitrified oocyte thaw cycles; (iii) oocyte or embryo donation cycles; and (iv) preimplantation genetic testing for monogenic disorders (PGT-M) or preimplantation genetic testing for structural rearrangements (PGT-SR) cycles as they refer to population with gene disorders or chromosomal abnormalities, instead of infertility. We further excluded planned fresh cycles that ended up with a frozen transfer (Supplementary Fig. S1). These included individuals were followed up to August 2023.

### Ovarian stimulation and endometrium preparation methods

Individuals underwent ovarian stimulation following one of two protocols: a gonadotrophin-releasing agonist (GnRH) (0.2 mg twice a day Synarel^®^ or 100 µg/day Decapeptyl, Pfizer Australia, West Ryde, Australia) or antagonist cycle (250 µg/day Orgalutran^®^ or Ganirelix, Merck Sharp & Dohme, Macquarie Park, Australia; 250 µg/day Cetrotide, Merck Serono, Frenchs Forest, Australia), with or without oral contraceptive pill scheduling (Levlen ED, Bayer Australia, Pymble, Australia), and with recombinant FSH (Gonal-F^®^ or Pergoveris, Merck Serono, Frenchs Forest, Australia; or Puregon or Elonva, Merck Sharp & Dohme, South Granville, Australia; or Menopur or Rekovelle^®^, Ferring Pharmaceuticals, Pymble, Australia; or Benfola, Gedeon Richter, North Sydney, Australia). Serum oestrogen (E2) was measured, and ultrasound monitoring began on day 5–7 of stimulation. A hCG trigger injection (250  or 500 µg recombinant Ovidrel, Merck Serono, Frenchs Forest, Australia; or 200 µg Decapeptyl, Ferring Pharmaceuticals, Pymble, Australia; or 5000 or 10 000 IU urinary Pregnyl, Organon, Lane Cove, Australia) was administered 35 or 36 h before the scheduled transvaginal oocyte retrieval.

In frozen embryo transfer cycles, the endometrium preparation method was either a natural cycle, a stimulation cycle, or a hormone replacement cycle. In natural FET cycles, no hormone was used during the endometrial preparation and ovulation was detected with a commercial urinary LH kit (Freedom Ovulation test kit, Freedom, Sydney, Australia), with subsequent confirmation of an LH surge. In stimulation cycles, 50 mg/day Clomiphene Citrate (ChemSupply, Port Adelaide, Australia) or 2.5 to 7.5 mg/day Letrozole (Novartis Pharmaceuticals, Macquarie Park, Australia) or recombinant FSH (Gonal-F^®^, Merck Serono, Frenchs Forest, Australia, or Puregon, Merck Sharp & Dohme, South Granville, Australia) was used. In hormone replacement cycles (with or without oral contraceptive pill and GnRH agonist down-regulation), additional oestrogen (2 mg/day oestradiol valerate, Advacare Pharma, Sydney, Australia, or 2 or 4 mg three times a day Progynova^®^, Bayer Australia, Pymble, Australia, or 2 or 4 mg three times a day Zumenon, Viatris Pty, Millers Point, Australia) was used and when the endometrial lining reached at least 6 mm, vaginal progesterone was given at 200 to 400 mg three times a day Urogestan (Besins Healthcare, Chatswood, Australia) or 400 mg/day Cyclogest (Gideon Richter, North Sydney, Australia) or 25 mg/day Prolutex (Boucher & Muir, North Sydney, Australia) or 80 µg twice daily or 250 µg Ovidrel (Merck Serono, Frenchs Forest, Australia).

### Oocyte retrieval and fertilization

Oocyte retrieval was undertaken 35 or 36 h after hCG trigger. Cumulus–oocyte complexes (COCs) were incubated in G-IVF PLUS (Vitrolife, Gothenburg, Sweden) after oocyte collection. Sperm preparation (swim-up or density gradient centrifugation) methodologies were employed, as described by Popkiss *et al.* (2022). At 40 h post-trigger, COCs were cultured with a prepared sperm sample in G-IVF PLUS (Vitrolife) for conventional IVF (standard insemination). Alternatively, 39 h after the ovulation trigger, COCs were denuded in 0.5 ml of 75 IU/ml hyaluronidase (Hyalase, Sanofiaventis, NSW, Australia) in G-IVF PLUS and then subjected to ICSI at 40–42 h post trigger, as previously detailed (Popkiss *et al.*, 2022). In brief, sperm were immobilized in a 10 μl PVP drop and aspirated before injection into mature metaphase-II (MII) oocytes. Inseminated oocytes were immediately moved to 25 μl G-1 PLUS drops under OVOIL in a microwell dish (Vitrolife), one oocyte per drop, for ongoing culture or in micro-well drops in an Embryoscope+ dish (Vitrolife). Oocytes were assessed for fertilization 16 to 18 h post insemination. All presumptive embryos were cultured at 37 °C in 6% CO_2_, 5% O_2_, 89% N_2_ in benchtop incubators (MINC, Cook Medical, Brisbane, Australia or PLANER, Origio, Malov, Denmark) or in an Embryoscope+ time-lapse incubator (Vitrolife).

### Embryo culture, vitrification, warming, and transfer

Embryos were cultured in sequential media (G-1 PLUS/G-2 PLUS, Vitrolife) and assessments were performed between day 2 and day 6. Blastocysts were developmentally classified (Weston *et al.*, 2009) and quality graded by assessing the trophectoderm and inner cell mass, as described previously (Gardner *et al.*, 2000), with the overall grade of each embryo (A, B, C, or D) corresponding to the lowest grade given, i.e.: trophectoderm or inner cell mass. Based on clinical prognosis, up to two embryos on day 5 or 6 were transferred. A single day 5 or 6 blastocysts was the preferred option, where possible. If embryos were suitable and PGT-A assessment was undertaken on day 5 or 6, then embryos were biopsied, as previously detailed (Cuman *et al.*, 2024). Biopsied and supernumerary embryos of suitable quality (C grade or above) were routinely vitrified on days 3, 5, and 6 immediately after assessment using a CVM CryoLogic vitrification method and the CMV Fibreplug cryodevice following the manufacturer’s instructions (CryoLogic, Blackburn, Australia) employing in-house media, based on SAGE vitrification and warming media formulations (SAGE™; CooperSurgical, Trumbull, CT, USA). Fresh or warmed embryos were transferred on day 3 or 5.

### Outcomes measures

The outcomes included: preterm birth (PTB, defined as gestational weeks < 37); low birth weight (LBW, defined as birth weight < 2500 g); high birth weight (HBW, defined as birth weight > 4000 g); small for gestational age (SGA, defined as birth weight < 10% for gestational age); large for gestational age (LGA, defined as birth weight > 90% for gestational age); and caesarean section. The birthweight percentiles were calculated based on the New Australian birthweight centiles, where intervention-initiated births were excluded and SGA and LGA were subsequently categorized (Joseph *et al.*, 2020). In addition, SGA and LGA based on the old birthweight centiles including all intervention-initiated births were also reported (Dobbins *et al.*, 2012).

### Statistical analyses

Continuous variables were summarized using means and SDs, and categorical variables were presented as event counts with corresponding percentages. Multivariable Poisson regression with robust variance was used to analyse all outcomes. Generalized estimating equations (GEEs) were used to account for the cluster effects of multiple embryo transfer cycles of the same individual. Both unadjusted and adjusted risk ratios (aRRs) with their corresponding 95% CI were reported. The adjusted model accounted for potential confounding factors, including female age, parity, semen source, ovulatory disorders (including polycystic ovary syndrome, PCOS), preimplantation genetic testing for aneuploidy (PGT-A), blastocyst transfer, number of embryos transferred, and site. As embryo quality has been shown not to be associated with perinatal outcomes, it was not included as a confounder in the main analysis (Zou *et al.*, 2023). The unit of analysis in GEE was individuals undergoing embryo transfer. We further compared frozen transfers with different endometrial preparation methods (natural, stimulation, and hormone replacement cycles) to the fresh transfers in a subgroup analysis.

A sensitivity analysis was also performed for individuals without ovulatory disorders to avoid potential bias arising from confounding by indication, as natural cycles are less likely to be offered to individuals with ovulatory disorders.

Another sensitivity analysis, including only blastocyst transfers, was conducted to investigate the robustness of the findings among blastocyst transfer cycles. In this analysis, intracytoplasmic sperm injection (ICSI) and blastocyst quality were included as additional confounders in the adjusted model.

All analyses were performed at STATA 18.0 (Stata/BE, StataCorp LLC, College Station, TX, USA). A *P*-value < 0.05 was considered statistically significant.

## Results

A total of 8081 women undergoing IVF who gave birth to 9243 newborns were included in this study. The detailed study population selection process is presented in Supplementary Fig. S1. Among these singletons, 3484 births were from the first included transfer cycle, and 5759 births were from the second to fifth embryo transfer cycles.

Cycle characteristics of individuals undergoing frozen versus fresh transfers are presented in Table 1. The mean age of individuals undergoing frozen transfer at oocyte retrieval was 34.0 ± 4.1 years. The majority of cycles involved used the partner’s semen for fertilization (88.1% of frozen cycles and 86.3% of fresh cycles) and blastocyst transfers (94.9% in frozen cycles and 81.9% in fresh cycles). Preimplantation genetic testing of aneuploidy was performed in 27.4% of frozen cycles. Prior to their IVF treatment, 4038 (65.9%) of individuals conceiving in frozen embryo transfer cycles and 2754 (88.3%) conceiving in fresh embryo transfer cycles were nulliparous. Among the frozen embryo transfer cycles, 3835 were natural cycles, 221 were ovarian stimulation cycles, and 2069 were hormone replacement treatment cycles.

**Table 1.** Cycle-based demographic and embryo transfer characteristics.

Characteristic		Frozen (n = 6125)	Fresh (n = 3118)	*P*-value*
Female age, mean (SD)		34.0 (4.1)	34.2 (4.2)	0.182
BMI		25.3 (5.5)	26.0 (5.8)	<0.001
Ovulation disorder (including PCOS)		1547 (25.3%)	654 (21.0%)	<0.001
Semen source	Partner	5393 (88.1%)	2690 (86.3%)	0.015
	Donor	732 (11.9%)	428 (13.7%)	
Parity	0	4038 (65.9%)	2754 (88.3%)	<0.001
	1	1903 (31.1%)	338 (10.8%)	
	2	166 (2.7%)	23 (0.8%)	
	3	18 (0.3%)	3 (0.1%)	
ICSI^#^	Yes	4740 (78.3%)	2424 (77.8%)	0.553
	No	1313 (21.7%)	693 (22.2%)	
SET	Yes	5757 (94.0%)	2744 (88.0%)	<0.001
	No	368 (6.0%)	374 (12.0%)	
Blastocyst transfer	Yes	5813 (94.9%)	2552 (81.9%)	<0.001
	No	312 (5.1%)	566 (18.1%)	
No. of transfer cycles	1	1395 (22.8%)	2089 (67.0%)	<0.001
	>1	4730 (77.2%)	1029 (33.0%)	
PGT	Yes	1677 (27.4%)	No	N/A
	No	4448 (72.6%)	No	
Endometrium prep.	HRT	2069 (33.8%)	N/A	N/A
	Natural	3835 (62.6%)	N/A	
	Stimulation	221 (3.6%)	N/A	

# 73 missing data in insemination.

* Chi^2^ test for categorical variables and continuous test for continuous variables. Statistical significance is defined as *P* < 0.05.

HRT, hormone replacement treatment; N/A, not applicable; PCOS, polycystic ovary syndrome; PGT, preimplantation genetic testing; prep, preparation; SET, single embryo transfer.

### Perinatal outcomes

The perinatal outcomes of frozen versus fresh embryo transfers are presented in Table 2.

**Table 2.** Association between frozen versus fresh embryo transfer and perinatal outcomes.

Outcome	**Fresh** (n = 3118)	**Frozen** (n = 6125)	Crude RR	**Adjusted RR** *
Caesarean section	1572 (50.4%)	3523 (57.5%)	1.14 (1.10–1.18)	1.14 (1.09–1.19)
Preterm birth	428 (13.7%)	547 (8.9%)	0.65 (0.58–0.73)	0.66 (0.58–0.76)
LBW	265 (8.5%)	322 (5.3%)	0.62 (0.53–0.72)	0.66 (0.55–0.78)
HBW	218 (7.0%)	621 (10.1%)	1.48 (1.28–1.73)	1.43 (1.21–1.68)
SGA	247 (7.9%)	257 (4.2%)	0.53 (0.45–0.63)	0.62 (0.51–0.75)
LGA	424 (13.6%)	1224 (20.0%)	1.49 (1.35–1.65)	1.37 (1.23–1.53)

* Poisson regression adjusted for female age, parity, ovulation disorder, semen source, preimplantation genetic testing for aneuploidy, blastocyst transfer, number of embryos transferred, and site.

Statistical significance is defined as *P *< 0.05.

HBW, high birth weight; LBW, low birth weight; LGA, large for gestational age; SGA, small for gestational age.

Compared with fresh transfers, frozen transfers were associated with a lower risk of preterm birth (8.9% vs 13.7%, aRR 0.66, 95% CI 0.58–0.76), LBW (5.3% vs 8.5%, aRR 0.66, 95% CI 0.55–0.78), and SGA (4.2% vs 7.9%, aRR 0.62, 95% CI 0.51–0.75), but a higher chance of caesarean section (57.5% vs 50.4%, aRR 1.14, 95% CI 1.09–1.19), HBW (10.1% vs 7.0%, aRR 1.43, 95% CI 1.21–1.68), and LGA (20.0% vs 13.6%, aRR 1.37, 95% CI 1.23–1.53).

Compared to the old birthweight centiles, there were fewer SGA (5.5% vs 7.3%) and more LGA (17.8% vs 12.0%) when the new birthweight centiles excluding all intervention-initiated births were used in the study population (Supplementary Table S1).

### Subgroup analysis

In the subgroup analysis, 2069 hormone replacement cycles and 3835 natural cycles were compared to fresh transfer cycles, respectively. Due to the small number of events of perinatal outcomes among individuals who had stimulation cycles, stimulation cycles were not included in this analysis (Table 3). We provided the results of stimulation cycles in Supplementary Table S2 for transparency, although the total number of events is small for all the outcomes except for caesarean section.

**Table 3.** Association between frozen embryo transfer with different endometrium preparation methods versus fresh embryo transfer and perinatal outcomes.

Outcomes	**Fresh** (n = 3118)	**Frozen** (n = 5904)^#^		Crude RR	Adjusted RR*
	Reference				
Caeserean section	1572 (50.4%)	1340 (64.8%)	HRT	1.25 (1.19–1.30)	1.24 (1.19–1.30)
		2060 (53.7%)	Natural	1.07 (1.03–1.12)	1.07 (1.02–1.12)
Preterm birth	428 (13.7%)	212 (10.3%)	HRT	0.74 (0.64–0.87)	0.75 (0.63–0.89)
		308 (8.0%)	Natural	0.58 (0.51–0.67)	0.59 (0.51–0.69)
LBW	265 (8.5%)	127 (6.1%)	HRT	0.72 (0.59–0.88)	0.77 (0.62–0.93)
		181 (4.7%)	Natural	0.55 (0.46–0.67)	0.59 (0.48–0.73)
HBW	218 (7.0%)	232 (11.2%)	HRT	1.63 (1.37–1.96)	1.45 (1.20–1.75)
		370 (9.7%)	Natural	1.40 (1.19–1.65)	1.42 (1.19–1.70)
SGA	247 (7.9%)	98 (4.7%)	HRT	0.60 (0.48–0.75)	0.72 (0.56–0.91)
		151 (3.9%)	Natural	0.50 (0.41–0.60)	0.56 (0.45–0.70)
LGA	424 (13.6%)	453 (21.9%)	HRT	1.63 (1.44–1.84)	1.38 (1.22–1.57)
		737 (19.2%)	Natural	1.43 (1.30–1.59)	1.36 (1.21–1.54)

* Poisson regression adjusted for female age, parity, PCOS, semen source, preimplantation genetic testing for aneuploidy, blastocyst transfer, number of embryos transferred, and site.

# Including 2069 HRT frozen cycles and 3835 natural frozen cycles. Due to the limited number of adverse events, mild stimulation cycles were not included in this analysis.

Statistical significance is defined as P < 0.05.

HBW, high birth weight; HRT, hormone replacement treatment; LBW, low birth weight; LGA, large for gestational age; Natural, natural cycle; SGA, small for gestational age.

Compared with fresh cycles, frozen transfers in hormone replacement cycles and natural cycles were both associated with a lower chance of PTB (hormone replacement cycle vs fresh: aRR 0.75, 95% CI 0.63–0.89; natural cycle vs fresh: aRR 0.59, 95% CI 0.51–0.69), LBW (hormone replacement cycle vs fresh: aRR 0.77, 95% CI 0.62–0.93; natural cycle vs fresh; aRR 0.59, 95% CI 0.48–0.73), and SGA (hormone replacement cycle vs fresh: aRR 0.72, 95% CI 0.56–0.91; natural cycle vs fresh: aRR 0.56, 95% CI 0.45–0.70). Similarly, compared with fresh transfers, frozen transfers with hormone replacement and natural cycles were both associated with a higher chance of caesarean section (aRR 1.24, 95% CI 1.19–1.30, aRR 1.07, 95% CI 1.02–1.12, respectively), HBW (aRR 1.45, 95% CI 1.20–1.75, aRR 1.42, 95% CI 1.19–1.70, respectively) and LGA (aRR 1.38, 95% CI 1.22–1.57, aRR 1.36, 95% CI 1.21–1.54, respectively).

### Sensitivity analysis

In the sensitivity analysis, 7042 singleton live births from individuals without ovulatory disorders were included. The findings were consistent with the main analysis: compared with fresh embryo transfer in ovulatory individuals, frozen embryo transfer in ovulatory individuals was associated with a reduced risk of PTB (aRR 0.68, 95% CI 0.58–0.80), LBW (aRR 0.68, 95% CI 0.55–0.83), and SGA (aRR 0.61, 95% CI 0.49–0.75), but an increased chance of caesarean section (aRR 1.14, 95% CI 1.09–1.20), HBW (aRR 1.41, 95% CI 1.17–1.70), and LGA (aRR 1.37, 95% CI 1.21–1.55) (Supplementary Table S3). The associations were mostly consistent when comparing hormone replacement cycles and natural cycles to fresh embryo transfer cycles (Supplementary Table S4).

Similarly, in another sensitivity analysis that only blastocyst transfers were included, frozen embryo transfer was associated with lower chance of PTB (aRR 0.67, 0.58–0.77), LBW (aRR 0.67, 0.56–0.81), and SGA (aRR 0.63, 0.52–0.78), but higher chance of HBW (aRR 1.42, 1.19–1.70) and LGA (aRR 1.38, 1.23–1.56), when ICSI and blastocyst quality were added as additional confounding factors (Supplementary Table S5). The associations remained robust when comparing hormone replacement cycles, natural cycles, and stimulation cycles to fresh embryo transfer cycles (Supplementary Table S2).

## Discussion

In comparison with fresh transfers, frozen transfers were associated with higher chances of caesarean section, and lower risks of PTB, LBW, and SGA, along with increased risks of HBW and LGA. The differences in these perinatal outcomes remained consistent in comparing the hormone replacement and natural cycles to the fresh cycles.

In this multicentre study, a large sample size of 9361 singleton live births based on routinely collected data from 12 IVF clinics was used for the analyses. With the clinical data, we were able to retrieve data on patients' characteristics and treatment protocol that was not available in previous national registry studies. Specifically, stratification of endometrium preparation methods allowed for comparisons with fresh embryo transfers. The adoption of vitrification as the sole freezing method also made the findings applicable to modern IVF practices and avoided the impact of freezing methods (e.g. slow-freezing) as a confounding factor. Furthermore, a set of prespecified confounding factors was selected in the adjusted regression model to reduce the risk of bias resulting from confounding. We additionally used the New Australian birthweight centiles to improve the accuracy and generalizability of the findings. Lastly, GEE models were used to account for the cluster effect of multiple cycles from the same couple.

However, some limitations should be noted. First, the retrospective design introduces inherent challenges of residual confounding. Second, the limited number of stimulation frozen embryo transfer cycles made the analysis of this subgroup impossible. Finally, due to the lack of information, we were unable to detect the impact of using versus not using the trigger in the natural cycles.

The findings in our study align with the findings from previous meta-analysis and some large cohorts, in that compared to fresh transfers, frozen transfers are associated with a lower risk of preterm birth, low birth weight and small for gestational age, but with a higher risk of high birthweight and large for gestational age (Kato *et al.*, 2012; Li *et al.*, 2014; Vidal *et al.*, 2017; Maheshwari *et al.*, 2018; Zhang *et al.*, 2018; Hwang *et al.*, 2019; Westvik-Johari *et al.*, 2021; Raja *et al.*, 2022; Choi *et al.*, 2025). Sibling analyses provide a unique opportunity by comparing outcomes of sibling births from the same mother and thereby removing time-invariant residual confounding, while they introduce potential selection bias. Sibling analyses from the UK and Nordic countries (Westvik-Johari *et al.*, 2021; Raja *et al.*, 2022) show higher large for gestational age rates in the frozen cycles, but this finding was not present in other cohorts (Ganer Herman *et al.*, 2022). This could be explained by the differences in the highly selected populations included in the sibling cohorts. Evidence from oocyte donation cycles provides additional insights by removing confounding factors relevant to embryo factors. The largest cohort study of 5848 singleton births from oocyte donation cycles showed similar birthweight and preterm birth rates between fresh or frozen cycles (Rafael *et al.*, 2022), while some earlier cohorts showed higher birthweights in the fresh cycles (Vidal *et al.*, 2017). These studies, together with our findings, revealed that the differences in perinatal outcomes between frozen and fresh transfers are unlikely due to the difference in the use of endometrial preparation methods in frozen cycles, but may be attributed to other factors including impaired endometrial characteristics, such as optimized endometrial receptivity in fresh cycles or the process of vitrification and warming embryos in frozen cycles.

Considering the underestimation of intra-uterine growth restriction resulting from the use of obstetrics interventions to deliver pre-term births in old birthweight centiles (Li *et al.*, 2014; Zhang *et al.*, 2018), we adopted the new centiles and observed fewer SGA babies and more LGA babies in our population (Supplementary Table S1). This reflects more accurate estimates of SGA and LGA in our population.

Existing evidence has shown that such a difference between perinatal outcomes following frozen versus fresh transfers could be due to multiple factors. Firstly, the methods of freezing, e.g. vitrification, may cause the difference. Vitrification, which involves ultra-rapid cooling and higher concentrations of cryoprotectants, has largely replaced slow-freezing due to its association with improved survival rates and clinical outcomes comparable to fresh embryo transfers (Vanderzwalmen *et al.*, 2013; Nagy *et al.*, 2020). Higher embryo survival rates after thaw (warming) and better pregnancy outcomes compared with slow-freezing are also reported (Debrock *et al.*, 2015; Kaye *et al.*, 2017). Clinics included in this study only used vitrification for embryo freezing, so we are unable to assess the differences between the two methods of freezing. Future research on this topic, when investigating historic datasets, could explore the differences between slow-freezing and vitrification. Secondly, the population undergoing these transfers, particularly women with polycystic ovary syndrome (PCOS), may influence outcomes. Our study has shown that individuals without PCOS undergoing frozen embryo transfer are still associated with reduced risk of preterm birth, low birth weight, and small for gestational age, but increased risk of high birth weight and large for gestational age. Though PCOS is associated with higher risks of hypertensive disorders during pregnancy and macrosomia, the differences in the perinatal outcomes between frozen and fresh embryo transfer are less likely to be caused by ovulatory disorder (Sindre *et al.*, 2023). Lastly, there is a discussion about whether endometrium preparation methods contribute to these differences. Different protocols for preparing the endometrium, such as natural versus hormone replacement cycles, could impact endometrial receptivity and subsequently affect perinatal outcomes (Roelens and Blockeel, 2025). In our study, the potential impact of endometrium preparation methods was evaluated in a subgroup analysis, but the results do not indicate that the differences are from these methods. Therefore, differences in perinatal outcomes after frozen and fresh transfer are more likely to result from the impaired endometrial characteristics in fresh cycles, the freezing technology, or the variations in populations. In practice, patients should be informed that the associated risk after fresh or frozen transfer and the risks are not driven by the endometrium preparation methods. Emerging evidence suggests that hormone replacement endometrium preparation may increase obstetric risks, especially preeclampsia (Pereira *et al.*, 2021), compared to natural cycles. This association appears to be independent of embryo vitrification, also likely due to the absence of corpus luteum in programmed cycles. The absence of functional corpus luteum could lead to decreased serum relaxin levels, potentially impairing the early gestational vascular remodelling (von Versen-Höynck *et al.*, 2020; Conrad *et al.*, 2022, 2024). While our subgroup analysis did not find significant differences in birthweight outcomes between hormone replacement cycles and natural cycles, literature suggests that maternal vascular outcomes may be related to status of corpus luteum rather than foetal growth metrics, which underscores the importance of considering the presence of corpus luteum in future studies evaluating perinatal outcomes following frozen transfer. Future research on frozen and fresh transfers, including those on long-term outcomes, should take endometrial preparation methods into consideration.

## Conclusions

Frozen embryo transfer is associated with a lower risk of preterm birth, low birth weight, and small for gestational age, compared with fresh embryo transfer. Such differences are unlikely due to the difference in the use of endometrial preparation methods in frozen cycles, but may be attributed to other factors, including impaired endometrial characteristics in fresh cycles or the process of vitrifying and warming embryos in frozen cycles.

## Supplementary Material

hoag002_Supplementary_Data

## Data Availability

The data underlying this article will be shared on reasonable request to the corresponding author, following the permission of Monash IVF Group.

## References

[hoag002-B1] Bergh PA , Navot D. The impact of embryonic development and endometrial maturity on the timing of implantation. Fertil Steril 1992;58:537–542. 1521649 10.1016/s0015-0282(16)55259-5

[hoag002-B2] Blockeel C , Campbell A, Coticchio G, Esler J, Garcia-Velasco JA, Santulli P, Pinborg A. Should we still perform fresh embryo transfers in ART? Hum Reprod 2019;34:2319–2329. 31803911 10.1093/humrep/dez233

[hoag002-B3] Chen ZJ , Shi Y, Sun Y, Zhang B, Liang X, Cao Y, Yang J, Liu J, Wei D, Weng N et al Fresh versus frozen embryos for infertility in the polycystic ovary syndrome. N Engl J Med 2016;375:523–533. 27509101 10.1056/NEJMoa1513873

[hoag002-B4] Choi SKY , Li W, Venetis C, Ledger W, Lui K, Harris K, Norman RJ, Jorm LR, Chambers GM. Perinatal risks associated with infertility and medically assisted reproduction: a population-based cohort study. Hum Reprod Open 2025;2025:hoaf020. 40453332 10.1093/hropen/hoaf020 PMC12124915

[hoag002-B5] Conrad KP , von Versen-Höynck F, Baker VL. Potential role of the corpus luteum in maternal cardiovascular adaptation to pregnancy and preeclampsia risk. Am J Obstet Gynecol 2022;226:683–699. 34437863 10.1016/j.ajog.2021.08.018

[hoag002-B6] Conrad KP , von Versen-Höynck F, Baker VL. Pathologic maternal and neonatal outcomes associated with programmed embryo transfer. J Assist Reprod Genet 2024;41:821–842. 38536594 10.1007/s10815-024-03041-9 PMC11052974

[hoag002-B7] Cuman C , Green MP, Willats E, Hardy T, Zander-Fox D. Variation in the incidence and type of full versus mosaic segmental aneuploidies identified in blastocysts undergoing PGT-A. FandR 2024;06:15–22.

[hoag002-B8] Debrock S , Peeraer K, Fernandez Gallardo E, De Neubourg D, Spiessens C, D’Hooghe TM. Vitrification of cleavage stage day 3 embryos results in higher live birth rates than conventional slow freezing: a RCT. Hum Reprod 2015;30:1820–1830. 26089301 10.1093/humrep/dev134

[hoag002-B9] Dobbins TA , Sullivan EA, Roberts CL, Simpson JM. Australian national birthweight percentiles by sex and gestational age, 1998–2007. Med J Aust 2012;197:291–294. 22938128 10.5694/mja11.11331

[hoag002-B10] Evans J , Hannan NJ, Edgell TA, Vollenhoven BJ, Lutjen PJ, Osianlis T, Salamonsen LA, Rombauts LJ. Fresh versus frozen embryo transfer: backing clinical decisions with scientific and clinical evidence. Hum Reprod Update 2014;20:808–821. 24916455 10.1093/humupd/dmu027

[hoag002-B11] Ganer Herman H , Mizrachi Y, Alon AS, Farhadian Y, Gluck O, Bar J, Kovo M, Raziel A. Obstetric and perinatal outcomes of pregnancies resulting from fresh versus frozen embryo transfer: a sibling cohort. Reprod Sci 2022;29:1644–1650. 35286664 10.1007/s43032-021-00570-x

[hoag002-B12] Gardner DK , Lane M, Stevens J, Schlenker T, Schoolcraft WB. Blastocyst score affects implantation and pregnancy outcome: towards a single blastocyst transfer. Fertil Steril 2000;73:1155–1158. 10856474 10.1016/s0015-0282(00)00518-5

[hoag002-B13] Hwang SS , Dukhovny D, Gopal D, Cabral H, Diop H, Coddington CC, Stern JE. Health outcomes for Massachusetts infants after fresh versus frozen embryo transfer. Fertil Steril 2019;112:900–907. 31466699 10.1016/j.fertnstert.2019.07.010 PMC6858957

[hoag002-B14] Joseph FA , Hyett JA, Schluter PJ, McLennan A, Gordon A, Chambers GM, Hilder L, Choi SK, de Vries B. New Australian birthweight centiles. Med J Aust 2020;213:79–85. 32608051 10.5694/mja2.50676

[hoag002-B15] Kato O , Kawasaki N, Bodri D, Kuroda T, Kawachiya S, Kato K, Takehara Y. Neonatal outcome and birth defects in 6623 singletons born following minimal ovarian stimulation and vitrified versus fresh single embryo transfer. Eur J Obstet Gynecol Reprod Biol 2012;161:46–50. 22200255 10.1016/j.ejogrb.2011.12.005

[hoag002-B16] Kaye L , Will EA, Bartolucci A, Nulsen J, Benadiva C, Engmann L. Pregnancy rates for single embryo transfer (SET) of day 5 and day 6 blastocysts after cryopreservation by vitrification and slow freeze. J Assist Reprod Genet 2017;34:913–919. 28500451 10.1007/s10815-017-0940-4 PMC5476550

[hoag002-B17] Li Z , Wang Y, Ledger W, Edgar D, Sullivan E. Clinical outcomes following cryopreservation of blastocysts by vitrification or slow freezing: a population-based cohort study. Hum Reprod 2014;29:2794–2801. 25316444 10.1093/humrep/deu246

[hoag002-B18] Maheshwari A , Pandey S, Amalraj Raja E, Shetty A, Hamilton M, Bhattacharya S. Is frozen embryo transfer better for mothers and babies? Can cumulative meta-analysis provide a definitive answer? Hum Reprod Update 2018;24:35–58. 29155965 10.1093/humupd/dmx031

[hoag002-B19] Nagy ZP , Shapiro D, Chang CC. Vitrification of the human embryo: a more efficient and safer in vitro fertilization treatment. Fertil Steril 2020;113:241–247. 32106970 10.1016/j.fertnstert.2019.12.009

[hoag002-B20] Pereira MM , Mainigi M, Strauss JF. Secretory products of the corpus luteum and preeclampsia. Hum Reprod Update 2021;27:651–672. 33748839 10.1093/humupd/dmab003 PMC8222764

[hoag002-B21] Popkiss S , Horta F, Vollenhoven B, Green MP, Zander-Fox D. Calcium chloride dihydrate supplementation at ICSI improves fertilization and pregnancy rates in patients with previous low fertilization: a retrospective paired treatment cycle study. J Assist Reprod Genet 2022;39:1055–1064. 35262809 10.1007/s10815-022-02407-1 PMC9107552

[hoag002-B22] Rafael F , Robles GM, Navarro AT, Garrido N, Garcia-Velasco JA, Bosch E, Nunes SG, Soares SR, Santos-Ribeiro S. Perinatal outcomes in children born after fresh or frozen embryo transfer using donated oocytes. Hum Reprod 2022;37:1642–1651. 35451027 10.1093/humrep/deac074

[hoag002-B23] Raja E-A , Bhattacharya S, Maheshwari A, McLernon DJ. Comparison of perinatal outcomes after frozen or fresh embryo transfer: separate analyses of singleton, twin, and sibling live births from a linked national in vitro fertilization registry. Fertil Steril 2022;118:323–334. 35717287 10.1016/j.fertnstert.2022.05.010

[hoag002-B24] Rienzi L , Gracia C, Maggiulli R, LaBarbera AR, Kaser DJ, Ubaldi FM, Vanderpoel S, Racowsky C. Oocyte, embryo and blastocyst cryopreservation in ART: systematic review and meta-analysis comparing slow-freezing versus vitrification to produce evidence for the development of global guidance. Hum Reprod Update 2017;23:139–155. 27827818 10.1093/humupd/dmw038 PMC5850862

[hoag002-B25] Roelens C , Blockeel C. Cycle management in frozen embryo transfer: the best of all worlds? Reprod Biomed Online 2025;50:104789. 40287192 10.1016/j.rbmo.2024.104789

[hoag002-B26] Roque M , Valle M, Guimarães F, Sampaio M, Geber S. Freeze-all policy: fresh vs. frozen-thawed embryo transfer. Fertil Steril 2015;103:1190–1193. 25747130 10.1016/j.fertnstert.2015.01.045

[hoag002-B27] Sindre HP , Westvik-Johari K, Spangmose AL, Pinborg A, Romundstad LB, Bergh C, Åsvold BO, Gissler M, Tiitinen A, Wennerholm UB et al Risk of hypertensive disorders in pregnancy after fresh and frozen embryo transfer in assisted reproduction: a population-based cohort study with within-sibship analysis. Hypertension 2023;80:e6–e16. 36154568 10.1161/HYPERTENSIONAHA.122.19689

[hoag002-B28] Smith A , Tilling K, Lawlor DA, Nelson SM. Live birth rates and perinatal outcomes when all embryos are frozen compared with conventional fresh and frozen embryo transfer: a cohort study of 337,148 in vitro fertilisation cycles. BMC Med 2019;17:202. 31718643 10.1186/s12916-019-1429-z PMC6852977

[hoag002-B29] Vanderzwalmen P , Connan D, Grobet L, Wirleitner B, Remy B, Vanderzwalmen S, Zech N, Ectors FJ. Lower intracellular concentration of cryoprotectants after vitrification than after slow freezing despite exposure to higher concentration of cryoprotectant solutions. Hum Reprod 2013;28:2101–2110. 23592220 10.1093/humrep/det107

[hoag002-B30] Vidal M , Vellvé K, González-Comadran M, Robles A, Prat M, Torné M, Carreras R, Checa MA. Perinatal outcomes in children born after fresh or frozen embryo transfer: a Catalan cohort study based on 14,262 newborns. Fertil Steril 2017;107:940–947. 28292612 10.1016/j.fertnstert.2017.01.021

[hoag002-B31] von Elm E , Altman DG, Egger M, Pocock SJ, Gøtzsche PC, Vandenbroucke JP, STROBE Initiative. The Strengthening the Reporting of Observational Studies in Epidemiology (STROBE) statement: guidelines for reporting observational studies. Int J Surg 2014;12:1495–1499.25046131

[hoag002-B32] von Versen-Höynck F , Häckl S, Selamet Tierney ES, Conrad KP, Baker VL, Winn VD. Maternal vascular health in pregnancy and postpartum after assisted reproduction. Hypertension 2020;75:549–560. 31838910 10.1161/HYPERTENSIONAHA.119.13779 PMC7491550

[hoag002-B33] Weston G , Osianlis T, Catt J, Vollenhoven B. Blastocyst transfer does not cause a sex-ratio imbalance. Fertil Steril 2009;92:1302–1305. 18996516 10.1016/j.fertnstert.2008.07.1784

[hoag002-B34] Westvik-Johari K , Romundstad LB, Lawlor DA, Bergh C, Gissler M, Henningsen AA, Håberg SE, Wennerholm UB, Tiitinen A, Pinborg A et al Separating parental and treatment contributions to perinatal health after fresh and frozen embryo transfer in assisted reproduction: a cohort study with within-sibship analysis. PLoS Med 2021;18:e1003683. 34170923 10.1371/journal.pmed.1003683 PMC8274923

[hoag002-B35] Zaat T , Zagers M, Mol F, Goddijn M, van Wely M, Mastenbroek S. Fresh versus frozen embryo transfers in assisted reproduction. Cochrane Database Syst Rev 2021;2:Cd011184. 33539543 10.1002/14651858.CD011184.pub3 PMC8095009

[hoag002-B36] Zhang B , Wei D, Legro RS, Shi Y, Li J, Zhang L, Hong Y, Sun G, Zhang T, Li W et al Obstetric complications after frozen versus fresh embryo transfer in women with polycystic ovary syndrome: results from a randomized trial. Fertil Steril 2018;109:324–329. 29338857 10.1016/j.fertnstert.2017.10.020

[hoag002-B37] Zhang Y , Fu X, Gao S, Gao S, Gao S, Ma J, Chen ZJ. Preparation of the endometrium for frozen embryo transfer: an update on clinical practices. Reprod Biol Endocrinol 2023;21:52. 37291605 10.1186/s12958-023-01106-5 PMC10249325

[hoag002-B38] Zou H , Kemper JM, Hammond ER, Xu F, Liu G, Xue L, Bai X, Liao H, Xue S, Zhao S et al Blastocyst quality and reproductive and perinatal outcomes: a multinational multicentre observational study. Hum Reprod 2023;38:2391–2399. 37877423 10.1093/humrep/dead212 PMC10694400

