# Supplementary material for: Frozen versus fresh embryo transfer on perinatal outcomes—do endometrial preparation methods matter?
Source: Hum Reprod Open. 2026 Jan 12;2026(1):hoag002. doi: 10.1093/hropen/hoag002 (PMC12848820; doi:10.1093/hropen/hoag002)
Supplement: hoag002_Supplementary_Data [file hoag002_supplementary_data.docx]

Number of cycles excluded

(n=13,124)

- AI (n=4,106)
- TI (n=760)
- TAFRO (n=446)
- Oocyte thaw cycle (n=1,080)
- Donor cycle (n=4,728)
- PGTM/SR (n=1,417)
- Frozen transfer in fresh cycles (n=579)
- Other (n=8)

Autologous frozen/fresh transfer cycles

- (n=56,810)

Total number of treatment cycles

- (n=69,934)

Cycles further excluded (n=46,099)

- Cancelled (n=17,505)
- No pregnancy (n=24,187)
- Pre-clinical (n=1,626)
- Miscarriage (n=2,646)
- Stillbirth (n=74)
- Inconclusive (n=22)
- Loss of follow-up (n=39)

Autologous frozen/fresh transfer cycles with singleton live births

- (10,253 cycles, 8,812 individuals)

Autologous frozen/fresh transfer cycles with live birth

- (n=10,711)

Autologous frozen/fresh transfer cycles with singleton live births

- (9,243 singleton live birth, 8,081 individuals)

Exclusion

- Multiple births (n=458)

Exclusion

- Missing of birth gender (n=968)
- Missing of birthweight (n=20)
- Gestational week <24 (n=22)

Fresh transfer

- (3,118 cycles, 3,041 individuals)

Frozen transfer

- (6,125 cycles, 5,494 individuals)

**Supplementary Figure S1. Study population selection process.**

AI: artificial insemination; TI: timed intercourse; TAFRO: embryo thaw, biopsy, and refreeze only

**Supplementary Table S1. Comparisons of small/large for gestational age between the new Australian birthweight centiles and the 2012 Australian birthweight centiles.**

| **Outcomes** | **Frozen** | **Fresh** | **Total** |
| --- | --- | --- | --- |
| **Number of cycles** | N=6,125 | N=3,118 | N=9,243 |
| **SGA^** | 257 (4.2%) | 247 (7.9%) | 504 (5.5%) |
|  |  |  |  |
|  |  |  |  |
| **SGA*** | 355 (5.8%) | 318 (10.2%) | 673 (7.3%) |
|  |  |  |  |
|  |  |  |  |
| **LGA^** | 1,224 (20.0%) | 424 (13.6%) | 1,648 (17.8%) |
|  |  |  |  |
|  |  |  |  |
| **LGA*** | 840 (13.7%) | 267 (8.6%) | 1,107 (12.0%) |

^birthweight percentiles calculated using the new Australia birthweight centiles

*birthweight percentiles calculated using the 2012 Australia birthweight centiles

**Supplementary Table S2. Association between frozen embryo transfer with different endometrium preparation methods versus fresh embryo transfer and perinatal outcomes in women undergoing blastocyst transfers**

| **Outcomes** | **Fresh** | **Frozen** | | **Crude RR** | **Adjusted RR** |
| --- | --- | --- | --- | --- | --- |
| **Number of cycles** | n=2,551 | n=5,736^#^ |  |  |  |
| **Cesarean section** |  |  |  |  |  |
|  | 1,266 (49.6%) | 1,292 (65.0%) | HRT | **1.26** (1.20-1.32) | **1.25**(1.19-1.32)* |
|  |  | 1,895 (53.6%) | Natural | **1.09** (1.04-1.14) | **1.08** (1.02-1.13)* |
|  |  | 116 (55.2%) | Stimulation | **1.17** (1.05-1.31) | **1.20** (1.07-1.34) |
| **Preterm birth** |  |  |  |  |  |
|  | 347 (13.6%) | 208 (10.5%) | HRT | **0.77** (0.65-0.90) | **0.76** (0.64-0.91)* |
|  |  | 282 (8.0%) | Natural | **0.58** (0.50-0.68) | **0.59** (0.50-0.69)* |
|  |  | 26 (12.4%) | Stimulation | 0.91 (0.63-1.32) | 0.96 (0.65-1.40) |
| **LBW** |  |  |  |  |  |
|  | 210 (8.2%) | 124 (6.2%) | HRT | **0.76** (0.61-0.94) | **0.78** (0.62-0.98)* |
|  |  | 167 (4.7%) | Natural | **0.57** (0.47-0.70) | **0.59** (0.47-0.74)* |
|  |  | 14 (6.7%) | Stimulation | 0.82 (0.48-1.40) | 0.84 (0.49-1.45) |
| **HBW** |  |  |  |  |  |
|  | 178 (7.0%) | 222 (11.1%) | HRT | **1.63** (1.34-1.97) | **1.43** (1.17-1.75)* |
|  |  | 343 (9.7%) | Natural | **1.41** (1.18-1.68) | **1.41** (1.16-1.71)* |
|  |  | 19 (9.1%) | Stimulation | 1.33 (0.85-2.08) | 1.31 (0.84-2.06) |
| **SGA** |  |  |  |  |  |
|  | 198 (7.8%) | 94 (4.7%) | HRT | **0.61** (0.48-0.77) | **0.73** (0.57-0.93)* |
|  |  | 139 (3.9%) | Natural | **0.51** (0.41-0.62) | **0.57** (0.45-0.73)* |
|  |  | 8 (3.8%) | Stimulation | **0.50** (0.25-0.98) | 0.63 (0.31-1.26) |
| **LGA** |  |  |  |  |  |
|  | 345 (13.5%) | 430 (21.6%) | HRT | **1.62** (1.42-1.84) | **1.39** (1.22-1.59)* |
|  |  | 687 (19.4%) | Natural | **1.45** (1.29-1.63) | **1.39** (1.22-1.59)* |
|  |  | 33 (15.7%) | Stimulation | 1.20 (0.86-1.66) | 1.21 (0.87-1.69) |

* adjusted for female age, parity, PCOS, semen source, ICSI, preimplantation genetic testing for aneuploidy, blastocyst quality, number of embryos transferred, site

HRT, hormone replacement treatment; Natural, natural cycle

^#^ including 1,989 HRT cycles, 3,537 natural frozen cycles, and 210 stimulation cycles

**Supplementary Table S3. Association between frozen versus fresh embryo transfer and perinatal outcomes in ovulatory women**

| **Outcomes** | **Fresh** | **Frozen** | **Crude RR** | **Adjusted RR** |
| --- | --- | --- | --- | --- |
| **Number of cycles** | N=2,464 | N=4,578 |  |  |
| **Cesarean section** | 1,246 (50.6%) | 2,659 (58.1%) | **1.15** (1.10-1.20) | **1.14** (1.09-1.20)* |
| **Preterm birth** | 337 (13.7%) | 425 (9.3%) | **0.68** (0.59-0.77) | **0.68** (0.58-0.80)* |
| **LBW** | 208 (8.4%) | 251 (5.5%) | **0.65** (0.54-0.77) | **0.68** (0.55-0.83)* |
| **HBW** | 166 (6.7%) | 447 (9.8%) | **1.48** (1.24-1.75) | **1.41** (1.17-1.70)* |
| **SGA** | 200 (8.0%) | 195 (4.3%) | **0.53** (0.43-0.64) | **0.61** (0.49-0.75)* |
| **LGA** | 328 (13.3%) | 891 (19.5%) | **1.48** (1.32-1.66) | **1.37** (1.21-1.55)* |

* adjusted for female age, parity, semen source, preimplantation genetic testing for aneuploidy, blastocyst transfer, number of embryos transferred, site

**Supplementary Table S4. Association between frozen embryo transfer with different endometrium preparation methods versus fresh embryo transfer and perinatal outcomes in ovulatory women**

| **Outcomes** | **Fresh** | **Frozen** | | **Crude RR** | **Adjusted RR** |
| --- | --- | --- | --- | --- | --- |
| **Number of cycles** | n=2,464 | n=4,457^#^ |  |  |  |
| **Cesarean section** |  |  |  |  |  |
|  | 1,246 (50.6%) | 862 (67.7%) | HRT | **1.29** (1.23-1.36) | **1.27**(1.21-1.36)* |
|  |  | 1,725 (54.2%) | Natural | **1.08** (1.03-1.13) | **1.07** (1.02-1.13)* |
| **Preterm birth** |  |  |  |  |  |
|  | 337 (13.7%) | 142 (11.2%) | HRT | **0.81** (0.67-0.97) | **0.79** (0.65-0.97)* |
|  |  | 268 (8.4%) | Natural | **0.61** (0.53-0.72) | **0.62** (0.52-0.74)* |
| **LBW** |  |  |  |  |  |
|  | 208 (8.4%) | 86 (6.8%) | HRT | 0.79 (0.62-1.01) | 0.80 (0.62-1.04)* |
|  |  | 158 (5.0%) | Natural | **0.59** (0.48-0.72) | **0.62** (0.50-0.78)* |
| **HBW** |  |  |  |  |  |
|  | 166 (6.7%) | 136 (10.7%) | HRT | **1.61** (1.30-2.01) | **1.47** (1.17-1.86)* |
|  |  | 302 (9.5%) | Natural | **1.43** (1.19-1.7) | **1.39** (1.14-1.70)* |
| **SGA** |  |  |  |  |  |
|  | 200 (8.1%) | 59 (4.6%) | HRT | **0.57** (0.43-0.76) | **0.65** (0.48-0.87)* |
|  |  | 132 (4.2%) | Natural | **0.51** (0.41-0.63) | **0.58** (0.46-0.74)* |
| **LGA** |  |  |  |  |  |
|  | 328 (13.3%) | 274 (21.5%) | HRT | **1.63** (1.41-1.88) | **1.41** (1.21-1.64)* |
|  |  | 598 (18.8%) | Natural | **1.43** (1.26-1.61) | **1.35** (1.18-1.56)* |

* adjusted for female age, parity, semen source, preimplantation genetic testing for aneuploidy, blastocyst transfer, number of embryos transferred, site

HRT, hormone replacement treatment; Natural, natural cycle

^#^ including 1,273 HRT cycles and 3,184 natural frozen cycles

**Supplementary Table S5. Association between frozen versus fresh embryo transfer and perinatal outcomes in women undergoing blastocyst transfers**

| **Outcomes** | **Fresh** | **Frozen** | **Crude RR** | **Adjusted RR** |
| --- | --- | --- | --- | --- |
| **Number of cycles** | N=2,551 | N=5,736 |  |  |
| **Cesarean section** | 1,266 (49.6%) | 3,303 (57.6%) | 1.16 (1.11-1.20) | 1.15 (1.10-1.20)* |
| **Preterm birth** | 347 (13.6%) | 516 (9.0%) | 0.66 (0.58-0.75) | 0.67 (0.58-0.77)* |
| **LBW** | 210 (8.2%) | 305 (5.3%) | 0.65 (0.55-0.76) | 0.67 (0.56-0.81)* |
| **HBW** | 178 (7.0%) | 584 (10.2%) | 1.48 (1.26-1.75) | 1.42 (1.19-1.69)* |
| **SGA** | 198 (7.8%) | 241 (4.2%) | 0.54 (0.45-0.65) | 0.63 (0.52-0.78)* |
| **LGA** | 345 (13.5%) | 1,150 (20.1%) | 1.50 (1.34-1.67) | 1.38 (1.23-1.56)* |

* adjusted for female age, parity, PCOS, semen source, ICSI, preimplantation genetic testing for aneuploidy, blastocyst quality, number of embryos transferred, site
